# Supracostal ultrasound guided approach percutaneous nephrolithotomy (SUGA-PNL) versus retrograde intrarenal surgery for large volume isolated upper calyceal stones: a prospective randomized analysis

**DOI:** 10.1007/s00240-024-01637-5

**Published:** 2024-10-29

**Authors:** Ahmed Assem, Ahmed Abdalla, Mohamed Elzoheiry, Islam Nasser Abd Elaziz, Hesham Amr, Heba Bakr, Ahmed M Rammah

**Affiliations:** 1 Department of Urology, Kasr Alainy hospitals, Faculty of Medicine, Cairo University, Cairo, Egypt; 2 Department of anesthesia, Kasr Alainy hospitals, Faculty of Medicine, Cairo University, Cairo, Egypt

**Keywords:** Upper calyceal stone, Supra-costal puncture, Ultrasound guided approach, Retrograde intrarenal surgery

## Abstract

To assess outcomes of supracostal ultrasound guided approach percutaneous nephrolithotomy (SUGA-PNL) and retrograde intrarenal surgery (RIRS) in isolated large volume upper calyceal stones (UCS). This was a prospective randomized study including patients with isolated UCS > 20mm. The patients were randomized into two groups: group (P) (SUGA-PNL) and group (R) (RIRS). Patients’ demographics, stones characteristics, operative, and postoperative outcomes essentially the stone free rate (SFR) and complications rate were documented. The stone clearance was defined as no fragments or residual fragments less than 2mm in the one month non contrast CT scan follow up. Eighty-nine patients opted to undergo the procedure according to the preoperative randomization. Four patients, 2 patients from each group, lost to follow up and other 2 patients were excluded from group (R) due to a tight ureter. Both groups were comparable as regards the preoperative demographics and stone characteristics. There were statistically significant differences regarding total operative time, the change in hemoglobin level, and postoperative pain score (*P*: 0.024, 0.010 and 0.032 respectively). The SFR was 88.1% in group (P) compared to 73.2% in group (R) (*P*: 0.019). Moreover, it did not differ significantly between both groups regarding the intraoperative and postoperative complications. No visceral and thoracic injuries were documented in group (P). On other side, 6 patients (14.6%) from group (R) had different grades of ureteral injury during access sheath placement. SUGA-PNL is a safe and effective treatment modality for UCS > 20mm with a higher SFR than RIRS.

## Introduction

Renal stone disease is a worldwide health problem. With an estimated prevalence of 10%, the incidence is globally increasing due to diabetes, obesity, and metabolic syndrome [1]. Over time, the management of renal stones has been converted from open surgery to percutaneous surgery, shock wave lithotripsy (SWL) and to minimally invasive procedures.

For many decades, percutaneous nephrolithotomy (PNL) has been widely accepted as the first treatment line for large renal calculi more than 20 mm. In the era of minimally invasive procedures, retrograde intrarenal surgery (RIRS) has come to the fore. For renal calculi less than 20 mm, RIRS has been mainly the first option [2]. Besides the technological advancements in flexible ureteroscopes and laser armamentarium, RIRS is increasingly adopted as an alternative treatment modality for large volume renal calculi, more than 20 mm, owing to its lower morbidity [3].

Ultrasound guided percutaneous access has been first introduced in Asia and Europe [4]. Since that time, many advantages have been attributed to its use in PNL such as limitation of radiation hazards and its lower cost compared to fluoroscopy [5]. Furthermore, the ultrasound allows visualization of the nearby viscera and assessment of vascularity through Doppler imaging [6].

Ever and anon, managing the isolated upper calyceal calculi could be difficult to decide. For renal calculi less than 20 mm, SWL and RIRS could be valid options while for more than 20 mm, PNL and RIRS would have pros and cons. Although providing an overall satisfactory stone free rate [7], these far-located stones could be unattainable through the traditional lower calyceal approach during PNL along with the associated morbidity. Moreover, RIRS could offer a warranted access, but such calculi of large volume might necessitate multiple sessions. Therefore, this study was designed to compare the outcomes (stone free rate and complications rate) of percutaneous nephrolithotomy (PNL) through direct ultrasound-guided upper calyceal approach and RIRS with laser lithotripsy in isolated upper calyceal stones more than 20 mm as a primary endpoint. The secondary endpoint was to assess the safety and feasibility of stent-less RIRS in large volume stones.

## Methods

This was a prospective randomized study that was carried out at Kasr Alainy hospitals, Cairo University between December 2022 and March 2024. The study was notarized by the research committee of ethics with code N-126. All participants were given a full elaboration on the study’s objectives and methods, and finally, a written consent was obtained from each participant.

The study comprised all patients aged ≥ 18 years old with isolated upper polar renal stones ≥ 20 mm in the maximal dimension while, patients with concomitant urinary tract stones or with prefixed ureteral stents or with active urinary tract infection were excluded. The participants were evaluated clinically, by full preoperative labs, urine analysis with treatment of infection if exists till clearance, and CT urography unless contraindicated. The patients underwent either percutaneous nephrolithotomy (group P) or retrograde intra-renal surgery with laser lithotripsy (group R) according to a preoperative computer-generated block randomization.

For group (P), after premedication with IV midazolam and pre-oxygenation at fraction of inspired oxygen (FIO_2_) 0.8, induction of general anesthesia was performed using propofol, fentanyl, and atracurium. Through the direct laryngoscopy, a double lumen endotracheal tube was inserted then checked by a fiberoptic bronchoscopy. During achieving the renal access, one lung ventilation (OLV), unless contraindicated, was conducted on a tidal volume of 6 ml/kg, respiratory rate at 16–20/min, and CO_2_ level up to 45mmHg. Before and after achieving the renal access, conventional two lung ventilation was performed. The patients were monitored thoroughly by electrocardiogram, pulse oximetry, capnography, and non-invasive blood pressure manometry.

**Fig. 1:**
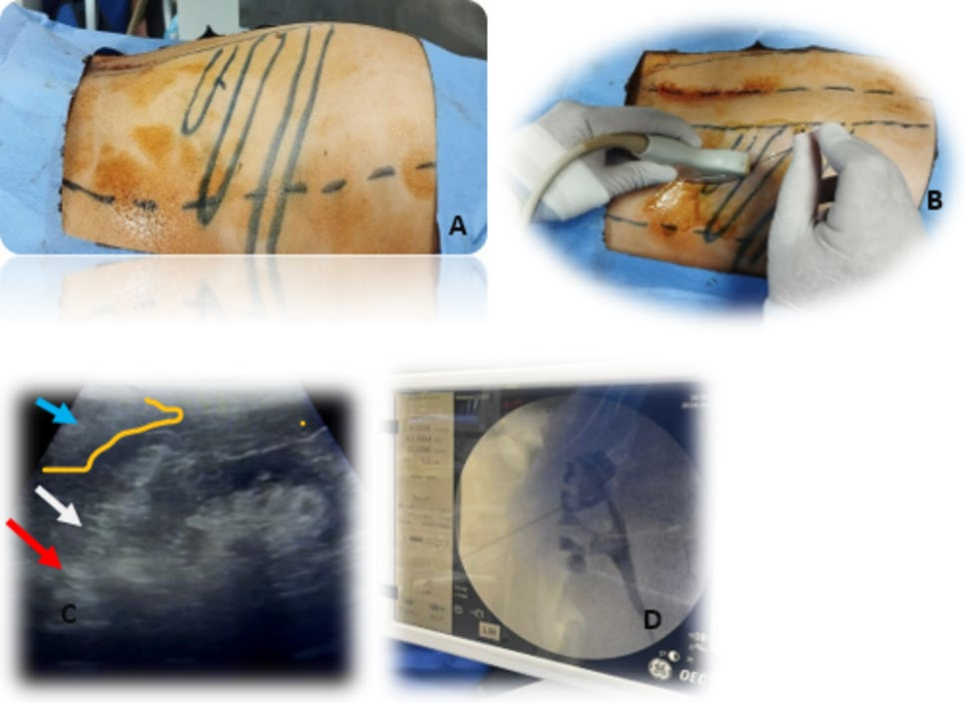
Demonstrates SUGA-PNL technique. **A**; shows the surface landmarks of last 3 ribs and the posterior axillary line, **B**; puncture needle placement supra-costal approach guided with ultrasound, **C**; ultrasound image shows the needle puncture through the targeted upper calyx harboring the stone, red arrow is the stone, white arrow is the puncture needle, blue arrow and yellow outline showed lung base, **D**: Fluoroscopic image of retrograde pyelography after needle placement in the targeted upper calyx. Notably, these figures are archived photos which were taken during the study, compiled by *Ahmed Assem*, ahmed.assem@cu.edu.eg, with all rights preserved

Under lithotomy position, cystoscopy and ipsilateral retrograde ureterography were carried out and an external ureteral catheter was inserted then the patients were put in the standard prone position. After surface marking of the last three ribs, paraspinal muscles, and the posterior axillary line, a 4.2 MHz abdominal transducer probe connected to a Siemens Acuson ultrasound (P500) was used to delineate the safe plane between the lung with its pleural covering, pleural recess and the upper pole of the kidney through the longitudinal plane approach. To preclude the renal parenchymal avulsion and pleural recess injury, the puncture site was always medial to the posterior axillary line and the puncture needle was directed to the calyx harboring the stone, targeted calyx, travelling through the renal parenchyma into the calyceal fornix, the renal papilla by the ultrasound view (Supracostal Ultrasound Guided Approach, SUGA). A J tip guidewire was then pushed gently through the targeted calyx to the renal pelvis. One cm transverse skin incision, at the lower half of the intercostal space and close to the puncture needle, was made then the tract was radially dilated by an Olbert balloon dilator (NephroMax 30Fr, inflated up to 20atm) and maintained by sliding an Amplatz sheath over the balloon, Fig. 1.

Through nephroscopy (KARL STORZ set, 26Fr operating sheath), the stones were disintegrated by pneumatic lithotripsy (Swiss LithoClast^®^, EMS) and retrieved by a nephroscopic forceps (KARL STORZ, 10.5Fr). The stone free status was checked by the nephroscopic vision and radiological control. Eventually, the Amplatz sheath, along with the external ureteral catheter, was removed without nephrostomy tube insertion. The indwelling jj stent was inserted only whenever indicated, i.e., inaccessible residual stones or pelvi-calyceal system injury. To assess the thoracic complications, an immediate postoperative chest x-ray was uniformly performed.

For group (R), under general anaesthesia, the patients underwent cystoscopy and ipsilateral ureterography in lithotomy position. A 7Fr (KARL STORZ) ureteroscope was advanced beside a previously passed guidewire to inspect the ureter and to assist the passive ureteral dilatation then a ureteral access sheath (UAS) (Navigator ^TM^ HD, 11/13Fr, 46 cm) was placed over the guidewire under fluoroscopic guidance. Ureteral tightness was considered if the small caliber ureteroscope could not be passed smoothly into the ureter and in such condition, the patient was ruled out. A single-use digital flexible ureteroscope (LithoVue^TM^, 9.5Fr, Boston Scientific) was advanced inside the UAS and laser lithotripsy (273 micron core diameter FlexiFib laser fibre connected to a high power Sphinx Holmium laser device, 80W) was implemented after stone identification through stone dusting (25–30HZ, 0.5J) alternating with stone fragmentation (5–8HZ, 2.5–3J) in a subjective continuum manner. A handful of stone fragments were retrieved by a Dormia basket (Zero Tip Nitinol Retrieval Basket, 3Fr, 120cm, Boston Scientific) for stone analysis. Lastly, the UAS was withdrawn under optical vision to inspect the entire ureter and the ureteral jj stent was only placed in case of significant ureteral injury in adherence to Traxer's Post Ureteroscopy Lesion Scale (PULSE, ≥ 3) [8].

The intraoperative parameters such as radiation time, total operative time, and irrigation fluid volume were monitored and compared between the two groups. In addition, the hospital stay, the catheterization time, and the pain score through the visual analogue scale (VAS) were documented. All intraoperative and postoperative complications were assessed and classified according to clavien-dindo scale. Postoperative systemic inflammatory response syndrome (SIRS) and sepsis were defined according to the National Institute for health and Care Excellence (NICE) guidelines [9].

During follow up, all patients were asked to visit the outpatient clinic after the first week of surgery then according to a scheduled regimen (at the 1^st^ month, 3^rd^ month and 6^th^ month after surgery). A non contrast (NC) CT scan was planned at the 1^st^ month visit to detect the stone free status where less than 2 mm residual stone was considered a stone free and the patients were then assessed for the need for auxiliary procedures. A urine analysis, kidney-bladder ultrasound, and serum kidney functions were also revised during the visits.

## Statistical analysis

Data were statistically characterized using the mean, standard deviation (SD), and range, or, when appropriate, frequencies (number of occurrences) and percentages. The paired t test was utilized to match the numerical data since the sample size was large enough, and the McNemar test was used to compare the categorical data. Through the study of the General Linear Model, Repeated Measure ANOVA was used for all comparisons of two variables across time among more than three time points. Statistics were regarded as significant when two-sided P values were < 0.05. IBM SPSS (Statistical Package for the Social Science; IBM Corp., Armonk, NY, USA) ver., twenty-two for Microsoft Windows was utilized to perform all statistical computations.

## Results

**Fig. 2:**
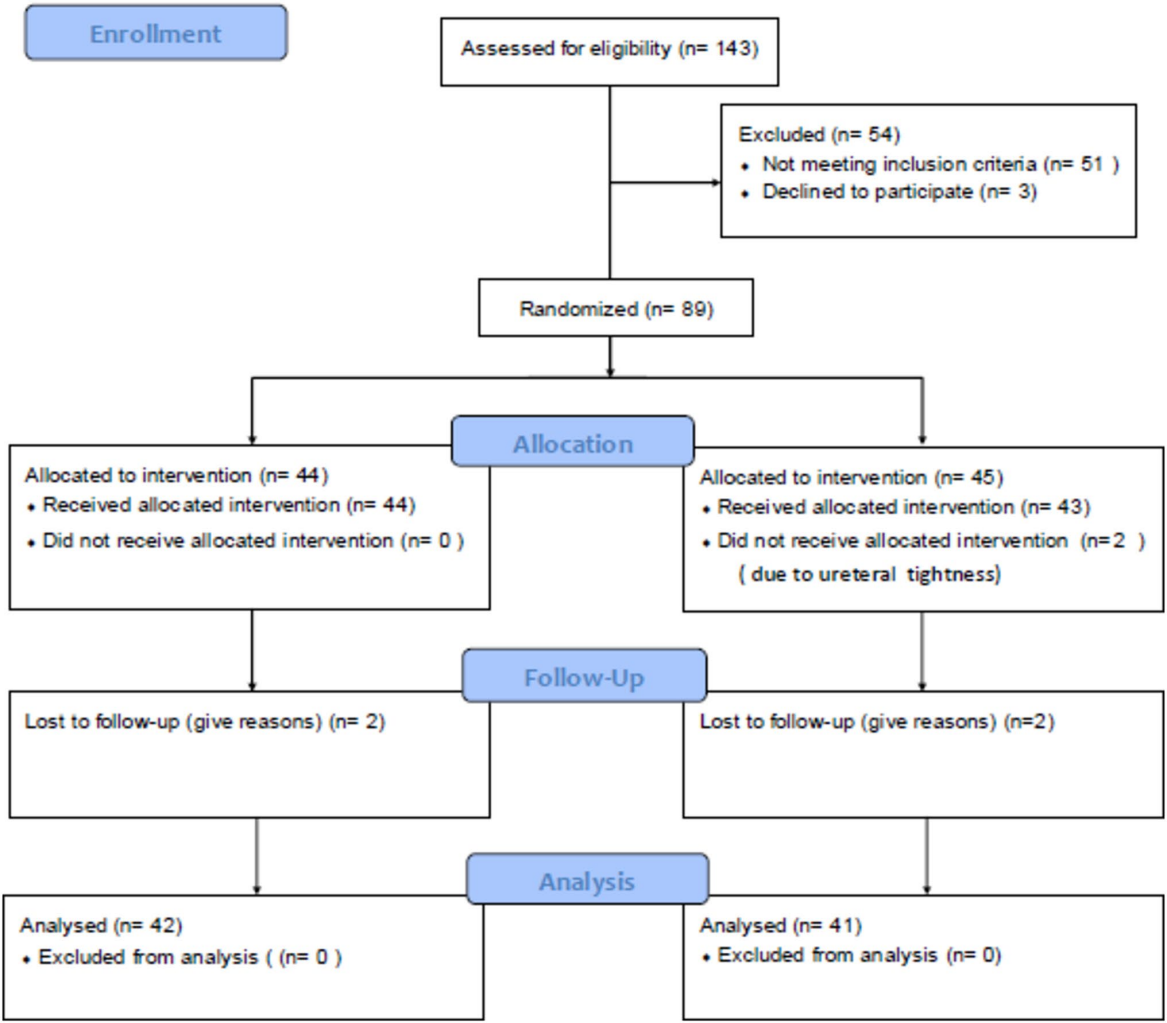
Flow chart illustrates patients' enrollment and allocation

One hundred and forty-three consecutive patients with isolated upper calyceal stones were screened from December 2022 to October 2023 and only 92 patients met the perquisite criteria. While 3 patients declined to participate, 89 patients opted to undergo the procedure according to the preoperative randomization. Two patients from group (R) were further excluded due to a non compliant ureter. Other 4 patients (2 patients from each group) lost to follow up. Therefore, 42 patients from group (P) and 41 patients from group (R) underwent the procedure and conformed to the follow up schedule, Fig. 2.

Both groups were balanced as regards the preoperative demographic or stone characteristics (all *P*-value ˃ 0.05), Tables 1, 2. While a significant statistical difference was reported between both groups regarding the irrigation fluid volume, the total operative time, the change in hemoglobin level, and postoperative VAS (*P* value: 0.012, 0.024, 0.010, and 0.032 respectively), it did not differ between both groups regarding the radiation time (*P* value = 0.521), Table 3. The jj ureteral stents were inserted in 3 patients in group (P) for inaccessible radiologically-visualized residual stones and in 2 patients in group (R) for UAS mediated injury during placement. Moreover, there was a substantial statistical difference between the two groups regarding the hospital stay and catheterization time (*P* value: 0.035 and 0.014 respectively), Table 3.

**Table 1:** Preoperative Demographic Data

Parameters	Group (P, PNL) (n = 42)	Group (R, RIRS) (n = 41)	*P*- value
Age (years)	40.5±17.5	42.5±19	0.926
Gender (n)			
Male	30	33	0.125
Female	12	8	
BMI	29.8±5.5	27.9±7.7	0.113
Comorbidities (n, %)			
DM	5 (11.9%)	3 (7.3%)	
HTN	3 (7.1%)	4 (9.8%)	
IHD	4 (9.5%)	2 (4.9%)	0.972
CKD	2 (4.8%)	3 (7.3%)	
Hyperparathyroidism	1(2.4%)	0	
Others	2 (4.8%)	3 (7.3%)	
Antiplatelet therapy	7 (16.7%)	5 (12.2%)	0.835
ASA Score			
Score 1	16 (38.1%)	14 (34.1%)	0.723
Score 2	20 (47.6%)	23 (56.1%)	
Score 3	6 (14.3%)	4 (9.8%)	

*BMI* body mass index, *DM* diabetes mellitus, *HTN* hypertension, *IHD* ischemic heart disease, *CKD* chronic kidney disease, *ASA score* American Society of Anesthesiologists score, 2020

**Table 2:** Stone Characteristics

Parameters	Group (P) (n = 42)	Group (R) (n = 41)	*P*- value
Laterality (n) Right Left	15 27	12 29	0.712
Density (HU)	1005.6±325.7	989.4±404.3	0.734
Maximal Dimension (mm)	31±10	29±9	0.998
Volume (mm^3^)	1587.5±500	1515.4±441	0.641
Area (mm^2^)	435±54	398±22	0.542
Number of stones			
Single	25 (59.5%)	27 (65.9%)	0.231
Multiple	17 (40.5)	14 (34.1%)	

*HU* Hounsfield unit

**Table 3:** Perioperative Data

Perioperative parameters	Group (P) (n = 42) mean±SD	Group (R) (n = 41) mean±SD	*P*- value
Radiation Time (minutes)	1.2± 0.6 min	1± 0.5	0.521
Irrigation Fluid Volume (liters)	13.5±4.2	3.5±2.6	0.012
Total Operative Time (minutes)	73.6±20.5	55.2±11.7	0.024
Preoperative Hemoglobin (gm/dl)	13.5±1.4	14±1.5	
Postoperative Hemoglobin (gm/dl)	12.6±0.8	13.8±1.2	0.010
Delta Hemoglobin	0.9±0.7	0.1±0.2	
Renal Access (n)			
Below the 12^th^ rib	4 (9.5%)		
Between the 12^th^ and 11^th^ rib	25 (59.5)		
Between the 11^th^ and 10^th^ rib	13 (31%)		
jj Stent Insertion (n)	3	2	1
Visual Analogue Scale (VAS)	5.5±3.4	2.6± 2.5	0.032
Hospital Stay (hours)	22.5±6	10±3.5	0.035
Catheterization Time (hours)	16.5±2	6±2.4	0.014

For group (P), the renal access was achieved through a supracostal approach in 90.5% of cases; the access was between the 11^th^ – 12^th^ in 59.5% of cases, and in 31% of cases between the 10^th^ – 11^th^ rib, while it was achieved through a subcostal approach in only 4 patients (9.5%) being of a relatively low- line kidney; all were by urologists.

As regards the stone free rate, 88.1% of group (P) compared to 73.2% of group (R) were stone free with a significant statistical difference between both groups (*P*-value = 0.019). Four patients (9.5%) from group (P) compared to 8 patients (19.5%) from group (R) had residual stones ˃ 4mm and a second session completion RIRS was required in 4 patients (9.8%) in group (R). One patient (2.4%) in group (P) and two patients (2.9%) in group (R) underwent conservative management for asymptomatic residual non-infectious stones upon shared patients' decision and no complications during the entire follow up were documented, Table 4.

**Table 4:** Stone Free Rated and Stone Analysis

	Group (P) (n = 42) (n, %)	Group (R) (n = 41) (n, %)	*P*- value
No residual stones	35 (83.3%)	28 (68.3%)	0.025
Less than 2 mm residual stone	2 (4.8%)	2 (4.9%)	0.999
Stone Free Rate	37 (88.1%)	30 (73.2%)	0.019
Residual Stone (2–4 mm)	1 (2.4%)	3 (7.3%)	
Residual stone more than 4 mm	4 (9.5%)	8 (19.5%)	
Retreatment methods			
PNL	0	0	
RIRS	1	4	
SWL	2	3	
Alkalinization therapy	1	2	
Watchful follow up	1	2	
Stone analysis			
Calcium oxalate monohydrate	29 (69.1%)	27 (65.9%)	0.932
Calcium oxalate dihydrate	10 (23.8%)	10 (24.4%)	
Calcium carbonate	2 (4.7%)	3 (7.3%)	
Uric acid	1(2.4%)	1(2.4%)	

*PNL* percutaneous nephrolithotomy, *RIRS* retrograde intrarenal surgery, *SWL* shockwaves lithotripsy

According to clavien dindo classification, a grade to comparable grade comparison was made and there was no statistically significant difference between both groups regarding the intraoperative or postoperative complications, Table 5. For group (P), 2 patients (4.8%) received hemotransfusion and one patient (2.4%) presented with urine leak from the puncture site, without a distal ureter obstruction by CT scan, and was managed conservatively. Neither thoracic nor visceral complications were documented in group (P). For group (R), 6 patients (14.6%) had different grades of ureteral injury during UAS placement. The injury site was in the distal ureter in all cases; 3 of them were of PULSE grade1-2, while the other three were of PULSE grade 3 for which a ureteral jj stent was inserted for 6 weeks with no add-on complications, namely ureteral stricture or secondary ipsilateral hydronephrosis, after stent removal during the follow up. Additionally, 4 patients (9.8%) in group (R) had frequent attacks of fever, Table 5. Only one patient (2.4%) in group (R) had developed urosepsis and required intensive care admission and was managed conservatively.

**Table 5:** Intraoperative and Postoperative Complications

	Group (P) (n = 42)	Group (R) (n = 41)	*P*- value
Grade (1)	1- Postoperative fever less than 38 managed conservatively without requiring antibiotics: (2) -(4.8%) 2- Urine leakage at the puncture site and managed by watchful waiting: (1) -(2.4%)	1- Ureteral injury (PULSE-1): (3) -(7.3%) 2- Ureteral injury (PULSE-2): (1) -(2.9%)	0.961
Grade (2)	1- Bleeding requiring blood transfusion: (2) -(4.8%) 2- Puncture site cellulites managed by antibiotics: (1) -(2.4%)	1- Ureteral injury (PULSE-3): (2)-(4.9%) 2- Postoperative fever less than 38 and required adding antibiotics at the ward: (2) -(4.9%)	0.782
Grade (3-A)	Postoperative fever (38.5) without urosepsis: (1)-(2.4%)	Postoperative fever (38.5) without urosepsis: (1) -(2.9%)	1
Grade (4-B)		Urosepsis requiring intensive care unit admission (ICU): (1) -(2.4%)	0.985

Three patients from group (P) compared to 2 patients from group (R) visited the hospital ER during the first 2 weeks of surgery (*P* value 0.941). For group (P), 2 patients had stent-related symptoms and one patient had a colicky pain for a passing ureteral stone (4mm in NCCT), whereas for group (R), one patient had stent-related symptoms and the other had a passing stone related colicky pain (3mm in NCCT). An alpha blocker (Tamsulosin 0.4 mg oral capsule) was prescribed and was reportedly effective as a medical expulsive therapy and in alleviating the stent-related symptoms.

## Discussion

On the whole, PNL is the gold standard treatment modality for renal stones more than 20 mm [10]; however, the management of large volume isolated upper calyceal stones represents a daunting challenge.

Nevertheless the subcostal approach to achieve the renal access during PNL is considered safe and satisfactory in most situations, the achievability to reach the upper calyceal stones from lower calyceal puncture could be low; 80% in supine position and merely 20% in prone position [11], besides the relatively longer access which is often associated with acute angle twist (torque), resulting in a calyceal neck injury with subsequent bleeding.

The prevailing view among many urologists is that the supracostal approach during PNL may be unfavorable being fraught with thoracic complications and higher incidence of primary bleeding. The following incidence of chest complications ranges from 10% (between the 11^th^ and 12^th^ ribs) to 35% (above the 11^th^ rib) [12] and may include lung organ injury and pleural injury with a secondary pneumothorax or effusion requiring a chest tube insertion. Apart from the possible crossing retropelvic artery, the upper polar renal puncture poses a special risk as the upper pole is almost wholly encompassed by large vessels [13, 14], which could make the entire procedure at stake.

During the study, the lung ipsilateral to the PNL side at the time of achieving the renal access was collapsed through OLV anaesthesia yielding a higher line lung base [15], hence providing a steady and relevantly wider ultrasound window between the upper pole of the kidney and the ipsilateral resting lung. Moreover, a transverse not longitudinal skin incision at the lower half of the intercostal space was made to spare the nearby intercostal vessels. Furthermore, the puncture needle was directed prudently, ultrasound- guided, traversing through the upper polar renal parenchyma into the renal papilla of the targeted calyx avoiding the renal infundibulum and its surrounding vessels injury. Therefore in the PNL group (P), no thoracic complications were documented during the study and only 2 patients with ischemic heart disease and a borderline preoperative hemoglobin required blood transfusion upon anaestheisa decision.

In line with a priori expectation, the RIRS (R) group had a significantly lower irrigation fluid volume, total operative time, decrease in hemoglobin level, postoperative VAS, and hospital stay than the PNL (P) group but the radiation time and grade to comparable grade complications rate were similar in both groups. The latter could be justified by the modification of the conventional supracostal approach into SUGA-PNL. Relatedly, Sahan and his colleagues retrospectively analyzed the upper polar puncture data under ultrasound versus fluoroscopic guidance and intimated that the radiation time and the hemoglobin drop were significantly lower in the ultrasound guidance group [16].

Contemporarily, some authors have assessed the outcome of RIRS in large volume renal stones (>20 mm). In a retrospective analysis comparing RIRS to PNL for renal stones (20–40 mm), Cosmin and his colleagues pointed out that RIRS is efficient as a treatment modality, however, the patients should be preoperatively counseled about the potential need for a second procedure to procure an admissible SFR [17]. Similarly, Elbakry concluded through a prospective study of 47 patients with large volume stones that RIRS could be offered as an alternative treatment for such stone volume albeit with an anticipated lower SFR even after a secondary procedure for stones >30 mm [3]. Consistently, in a meta- analysis comparing the different treatment modalities of renal stones, PNL had the highest SFR and RIRS had the lowest SFR with the lowest rank by the rank probability test [18].In the current study, the RIRS (R) group had a significantly lower SFR compared to the PNL group (*P* value = 0.019) and 4 patients (9.8%) required a second session completion RIRS (1.1 RIRS procedure/patient).

Ureteral stenting after RIRS is considered a routine practice [19]. According to the anecdotal evidence among urologists, stenting post RIRS has a reparatory role in ureteral stricture prevention after UAS deployment and to prevent the emergence of pain during passage of stone fragments [20]. A few studies in literature looked into the feasibility of stent-less RIRS. Based on a randomized prospective analysis, Sirithanaphol and his colleagues found it safe not to stent the ureter after uneventful RIRS [21]. Cesar et al. concluded that omission of ureteral stenting for uncomplicated pre-stented RIRS cases is feasible, even though ureteral stenting might alleviate the postoperative pain [22]. During the study, ureteral stenting was reserved for those with significant UAS mediated ureteral injury although none of these cases were preoperatively stented. For the whole arm (R), the ureteral stricture was not encountered during the 6 months follow up.

According to what the authors know, this is first piece of research that fully describes the ultrasound guided upper calyceal approach combined with OLV anaesthesia and to assess the feasibility of the stent-less RIRS after large volume renal stones laser lithotripsy. Regretfully, due to the paucity of isolated upper calyceal stones of large volume, the study was limited by the small sample size. Thus, future studies of a larger scale and longer follow up are required to emphasize the study's results.

## Conclusion

With a minimal postoperative morbidity, supracostal ultrasound guided approach percutaneous nephrolithotomy (SUGA-PNL) provides a higher stone free rate than retrograde intrarenal surgery with laser lithotripsy (RIRS) apropos management of isolated upper calyceal stones more than 20 mm.

## Data Availability

Sequence data that support the findings of this study have been deposited in Kasr Alainy hospital Archive.

## Abbreviations

SUGA-PNL: Supracostal Ultrasound Guided Approach Percutaneous Nephrolithotomy
RIRS: Retrograde Intrarenal Surgery
UCS: upper calyceal stone
NC-CT: Non contrast computer tomography
SFR: Stone free rate
SWL: Shock Waves Lithotripsy
